# IgA Nephropathy Is the Most Common Underlying Disease in Patients With Anticoagulant-Related Nephropathy

**DOI:** 10.1016/j.ekir.2022.01.1048

**Published:** 2022-01-19

**Authors:** Hernando Trujillo, Justo Sandino, Teresa Cavero, Fernando Caravaca-Fontán, Eduardo Gutiérrez, Ángel M. Sevillano, Amir Shabaka, Gema Fernández-Juárez, Pablo Rodríguez Doyágüez, Rocío Gimena Muñoz, Leonardo Calle García, Virginia Cabello, José Manuel Muñoz-Terol, Ana García Santiago, Oscar Toldos, Juan Antonio Moreno, Manuel Praga

**Affiliations:** 1 Department of Nephrology, Hospital Universitario 12 de Octubre, Madrid, Spain; 2 Instituto de Investigación del Hospital Universitario 12 de Octubre (imas12), Madrid, Spain; 3 Department of Nephrology, Hospital Universitario Fundación Alcorcón, Madrid, Spain; 4 Department of Nephrology, Hospital Universitario Príncipe de Asturias de Alcalá de Henares, Madrid, Spain; 5 Department of Nephrology, Complejo Asistencial de Segovia, Segovia, Spain; 6 Department of Nephrology, Hospital Universitario Virgen del Rocío, Sevilla, Spain; 7 Department of Nephrology, Hospital Universitario Marqués de Valdecilla, Santander, Spain; 8 Department of Pathology, Hospital Universitario 12 de Octubre, Madrid, Spain; 9 Department of Cell Biology, Physiology and Immunology, University of Cordoba, Maimonides Biomedical Research Institute of Cordoba (IMIBIC), UGC Nefrología, Hospital Universitario Reina Sofía, Córdoba, Spain; 10 Department of Medicine, Universidad Complutense de Madrid, Madrid, Spain

**Keywords:** acute kidney injury, anticoagulation, hematuria, IgA nephropathy, kidney biopsy

## Abstract

**Introduction:**

Anticoagulant-related nephropathy (ARN) is a relatively novel recognized entity characterized by hematuria-associated acute kidney injury (AKI) in the context of overanticoagulation. Preexisting or underlying kidney disease seems to be a predisposing factor; however, few studies have described histologic findings in patients with ARN. We aimed to evaluate underlying kidney pathology in patients on oral anticoagulation who presented an episode of AKI with hematuria in whom a kidney biopsy was performed.

**Methods:**

Retrospective observational multicenter case study in patients treated with oral anticoagulants who developed macroscopic or intense hematuria followed by AKI. Only patients with available kidney biopsy specimens were included. Histologic findings and clinical data throughout follow-up were analyzed.

**Results:**

A total of 26 patients were included with a median age of 75 years (62–80) and a follow-up period of 10.1 months. Of the patients, 80% were male, and most cases (92%) were on anticoagulation with vitamin K antagonists (VKAs). At admission, median serum creatinine (SCr) level was 4.2 mg/dl (2.8–8.2), median international normalized ratio (INR) 2.4 (1.5–3.4), and 11 patients (42%) required acute dialysis during hospitalization. Kidney biopsy results revealed that all patients except 1 had an underlying nephropathy: IgA nephropathy (IgAN) in 19, probable IgAN in 1, diabetic nephropathy in 3, nephrosclerosis in 1, and idiopathic nodular glomerulosclerosis in 1. At 12 weeks after discharge, only 6 subjects (24%) attained complete kidney recovery whereas 7 (28%) remained on chronic dialysis.

**Conclusion:**

IgAN was the most common underlying kidney disease in our biopsy-proven series of ARN, in which a significant percentage of patients did not achieve kidney function recovery.

## Graphical abstract

**Figure:**
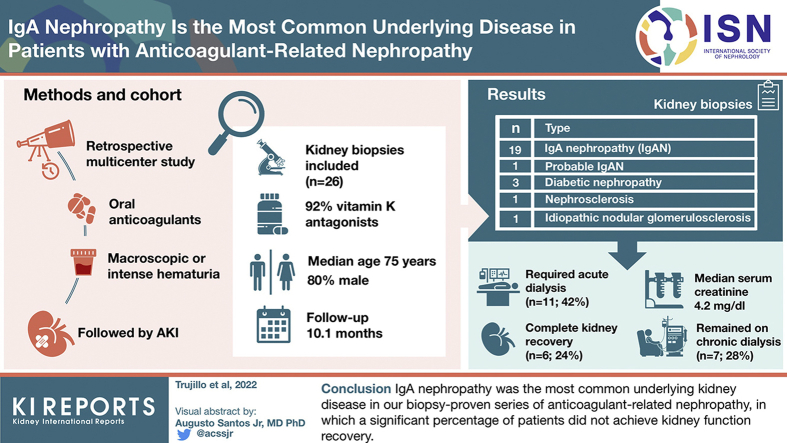

ARN was originally described in 2009 in a small case series of patients on oral anticoagulation with warfarin and unexplained AKI plus microscopic or gross hematuria.^1^ Although VKAs were the first drugs associated with this entity, it is now recognized that other agents, such as direct-acting oral anticoagulants (DOACs), antiplatelet medications, heparin, or even prohemorrhagic states, may trigger a similar clinical picture.^2^^,^^3^ Histologically, ARN is characterized by glomerular hemorrhage and acute tubular injury with profuse red blood cells (RBCs) and hematic casts at the tubular level. To date, only 3 case series and several case reports have included detailed descriptions of kidney biopsies in patients with ARN, where most cases had an underlying kidney disease. Interestingly, IgAN was frequently encountered in early reports^1^^,^^4^ and IgA-predominant immune complexes were observed in 30% to 40% of patients in the largest and most recent series.^3^ In general, kidney survival after ARN is poor, with most patients exhibiting only partial recovery of kidney function or remaining on dialysis.^1^^,^^4^ The main predisposing factors for ARN include overanticoagulation, older age, and preexisting nephropathy^5^; nonetheless, features associated with kidney recovery have been less explored. Little is known on the optimal therapy for ARN, which currently remains largely supportive, involving anticoagulation reversal or correction of baseline coagulopathy; however, it is unclear whether this may be effective.

The aims of this work were to describe histologic findings in kidney biopsies of patients with clinical suspicion of ARN, to evaluate renal prognosis, and to perform a thorough review of histopathologic findings in patients with ARN reported in the literature.

## Methods

### Study Population

A total of 26 patients referred to 6 nephrology departments belonging to the Spanish Group for the Study of Glomerular Diseases between 1996 and 2019 were retrospectively studied. We included adult patients on chronic oral anticoagulant therapy with VKAs or DOACs who presented an episode of gross hematuria or intense microhematuria on urinary sediment followed by AKI (regardless of INR value) in which a kidney biopsy was performed. Patients without histologic assessment were excluded. All patients were admitted to the hospital during the anticoagulation-related AKI episode and were discharged according to the criteria of the treating physician.

This study was performed in accordance with the Declaration of Helsinki. Given the retrospective nature of the study, a waiver of informed consent from individual patients was granted.

### Clinical, Laboratory, and Histopathologic Data

Baseline and follow-up data were compiled from medical records of all participating centers, following a uniform protocol that included demographics, clinical presentation, therapeutic management, pathologic features of kidney biopsy specimens, and laboratory parameters that were deemed of interest.

In order to assess reproducibility of biopsy evaluations by pathologists, 22 of the 26 (84.6%) kidney biopsy specimens were centrally assessed at Hospital Universitario 12 de Octubre by an expert pathologist (OT) who was blinded to clinical data. The following pathologic features were recorded by light microscopy: number of glomeruli per biopsy, percentage of glomerulosclerosis, mesangial expansion (graded as none to diffuse), endocapillary proliferation, extracapillary proliferation, focal segmental glomerulosclerosis lesions, interstitial fibrosis and tubular atrophy (graded as none to severe), arterio- and arteriolosclerosis (presence of intimal thickening ≥ thickness of media), RBC casts in tubules, and acute tubular necrosis. Except for 1 case, all samples were further evaluated with direct immunofluorescence staining with IgA, IgM, IgG, C3, C4, C1q, Igκ, and Igλ (when available) (graded as 0–3+). In cases where sufficient material was available, an electron microscopy study was also conducted. Furthermore, we applied a chronicity score that has been validated for C3 glomerulopathy.^6^^,^^7^

### Definitions and Outcomes

The definition and staging of AKI were described according to the Kidney Disease: Improving Global Outcomes guidelines.^8^ Baseline kidney function was defined as the estimated glomerular filtration rate (eGFR) value 12 weeks before hospital admission. Final kidney function was defined as the eGFR (Modification of Diet in Renal Disease study equation) value obtained 12 weeks after hospital discharge. Complete recovery was defined as recovery of at least 90% of baseline eGFR. No recovery was defined by an eGFR value that was lower than at least 25% of the baseline value or the need for chronic dialysis.

The main outcome was to describe pathologic findings in kidney biopsy specimens of patients with clinical suspicion of ARN. Secondary outcomes were to evaluate renal prognosis during follow-up and to analyze histopathologic findings in patients with ARN previously reported by means of a thorough literature review.

### Search Strategy and Selection Criteria

Given the scarcity of published data regarding biopsy-proven ARN, a thorough review of the literature was conducted to perform a comparative analysis of our data. We searched PubMed, the Cochrane Library, MEDLINE, and reference lists from relevant articles up to and including August 1, 2021. We used the search terms “anticoagulant-related nephropathy,” “warfarin-related nephropathy,” “hematuria and anticoagulant treatment,” “IgA nephropathy and anticoagulation,” OR “acute kidney injury” in combination with the terms “anticoagulation,” OR “anticoagulant therapy,” OR “anticoagulant treatment,” OR “warfarin,” OR “vitamin K antagonists,” OR “novel oral anticoagulants,” OR “hematuria,” OR “gross hematuria,” OR “macrohematuria.” Only English language papers were reviewed. This search revealed 3 case series^1^^,^^3^^,^^4^ and 29 case reports,^2^^,^^9^, ^10^, ^11^, ^12^, ^13^, ^14^, ^15^, ^16^, ^17^, ^18^, ^19^, ^20^, ^21^, ^22^, ^23^, ^24^, ^25^, ^26^, ^27^, ^28^, ^29^, ^30^, ^31^, ^32^, ^33^, ^34^, ^35^ comprising a total of 92 further cases of adult patients with biopsy-proven ARN.

### Statistical Analysis

Results are expressed as frequencies and percentages for categorical variables and medians and interquartile ranges (IQRs) for continuous variables. Proportions were compared with the use of the Fisher exact probability test. Mann-Whitney *U* test was used for comparing medians. *P* < 0.05 were established as criteria for statistical significance. All statistical tests were 2 sided and performed with Stata software for Windows, version 12.0 (StataCorp LP, College Station, TX).

## Results

### Patients

The study comprised a total of 26 patients with a median age of 75 years (IQR 62–80), 80% male. Most patients (92%) had hypertension, 38% diabetes, and 42% chronic kidney disease (CKD). Baseline median eGFR and SCr were 62 ml/min per 1.73 m^2^ (IQR 49–75) and 1.1 mg/dl (IQR 0.9–1.4), respectively. Of 11 cases with urine sediment available in the previous 6 months before the AKI episode, 8 (72%) already had microhematuria, of whom 6 (54%) presented >20 RBCs per high-power field. VKAs represented the most frequently used anticoagulant agent (92% of the cases) with a median length of anticoagulation therapy of 48 months (IQR 12–89) and a median INR at the time of hospital admission of 2.4 (IQR 1.5–3.4). Clinical presentation was characterized by gross hematuria in 84% of the cases, intense microhematuria in the rest, and all exhibited AKI with a median peak SCr of 6.3 mg/dl (IQR 3.8–9.5). Most patients presented with AKI stage 3 (84.6%), 11.5% with AKI stage 2, 3.8% with AKI stage 1, and 42% required acute dialysis. Additional management included anticoagulation withdrawal in 61% and immunosuppression based on corticosteroids in 73% and mycophenolic acid in 19%. The main clinical and laboratory features of the population are detailed in Table 1.

**Table 1:** Clinical characteristics of the study population and kidney outcomes according to kidney pathology

Variable	*N =* 26	*n =* 20	*n =* 6	*P*
Baseline	All patients	IgAN	Non-IgAN diseases	
Sex, *n* (%), male	21 (80)	15 (75)	6 (100)	0.29
Age, Md (IQR), yr	75 (62–80)	75 (63–80)	72 (54–89)	0.71
BMI, Md (IQR), kg/m^2^	29.8 (25.7–32.4)	29.9 (25.4–32.4)	26.4 (26.4)	0.73
HTN, *n* (%)	24 (92.3)	18 (90)	6 (100)	1
DM, *n* (%)	10 (38.5)	7 (35)	3 (50)	0.64
Hepatic cirrhosis, *n* (%)	3 (11.5)	2 (10)	1 (16.7)	1
Chronic kidney disease, *n* (%)	11 (42.3)	7 (35)	4 (66.7)	0.62
SCr, Md (IQR), mg/dl^a^	1.1 (0.9–1.4)	1.1 (0.9–1.4)	1.3 (1.2–1.4)	0.69
eGFR, Md (IQR), ml/min per 1.73 m^2^	62 (49–75)	66 (49–81)	56 (48–62)	0.48
Indication for anticoagulant therapy, *n* (%)				0.78
Arrhythmia	18 (69.2)	14 (70)	4 (66.7)	
Thrombotic event	6 (23.1)	4 (20)	2 (33.3)	
Prosthetic heart valve	2 (7.7)	2 (10)	0 (0)	
Type of anticoagulant, *n* (%)				0.41
Vitamin K antagonists	24 (92.3)	19 (95)	5 (83.3)	
Direct-acting oral anticoagulants	2 (7.7)	1 (5)	1 (16.7)	
Anticoagulation length, Md (IQR), mo	48 (12–89)	44 (12–84)	76 (13–152)	0.37
Hospital admission				
Gross hematuria, *n* (%)	22 (84.6)	19 (95)	3 (50)	0.02
SCr, Md (IQR), mg/dl	4.2 (2.8–8.2)	4.0 (2.8–8.2)	4.6 (2.7–13.2)	0.63
Peak SCr, Md (IQR), mg/dl	6.3 (3.8–9.5)	6.3 (3.8–8.6)	8.1 (3.8–14.1)	0.54
Acute dialysis, *n* (%)	11 (42.3)	8 (40)	3 (50)	1
INR, Md (IQR)	2.4 (1.5-3.4)	2.5 (1.5–3.3)	2.1 (1.4–5.1)	0.89
Anticoagulation withdrawal, *n* (%)	16 (61.5)	10 (50)	6 (100)	0.053
Anticoagulation restart (at discharge), *n* (%)	12 (75)	8 (40)	4 (66.7)	0.60
Immunosuppressive treatment (for AKI), *n* (%)	19 (73.1)	17 (85)	2 (33.3)	0.028
Corticosteroids, *n* (%)	19 (73.1)	17 (85)	2 (33.3)	0.028
Mycophenolic acid, *n* (%)	5 (19.2)	5 (25)	0 (0)	0.54
Kidney function recovery^b^				
Complete recovery, *n* (%)	6 (24)	5 (26.3)	1 (16.7)	1
No recovery, *n* (%)	19 (76)	14 (73.7)	5 (83.3)	1
Chronic dialysis, *n* (%)	7 (28)	5 (26.3)	2 (33.3)	1
Baseline SCr	1.1 (0.9–1.4)	1.1 (0.9–1.4)	1.3 (1.2–1.4)	0.69
Baseline eGFR	62 (49–75)	66 (49-81)	56 (48–62)	0.48
Final SCr	2.2 (1.8–4.5)	2.2 (1.7–4.5)	3.0 (1.9–13)	0.56
Final eGFR	23 (14–39)	25 (14–39)	20 (4–39)	0.48

AKI, acute kidney injury; BMI, body mass index; DM, diabetes mellitus; eGFR, estimated glomerular filtration rate; HTN, hypertension; IgAN, IgA nephropathy; INR, international normalized ratio; IQR, interquartile range; Md, median; MDRD, Modification of Diet in Renal Disease; SCr, serum creatinine.

Continuous data are expressed as Md (IQR), categorical variables as *n* (%). eGFR was calculated by MDRD study equation.

a SCr available in the last 12 weeks before admission.

b Evaluated at 12 weeks after hospital discharge (missing data in 1 patient).

### Histopathologic Findings

All patients underwent a kidney biopsy as a consequence of the AKI episode, with a median time to kidney biopsy from admission of 14 days (IQR 5–30). Except for 1 case, an underlying nephropathy was found in every sample (Table 2). IgAN was found in 19 cases (73%), probable IgAN (mesangial expansion in light microscopy plus mesangial deposits in electron microscopy; immunofluorescence study not performed because of technical problems) in 1 (3.8%), diabetic nephropathy in 2 (7.7%), diabetic nephropathy plus nephrosclerosis in 1 (3.8%), nephrosclerosis in 1 (3.8%), and idiopathic nodular glomerulosclerosis in 1 (3.8%). Tubules filled with RBC casts and acute tubular necrosis were observed in 80% and 88% of the cases, respectively, and most specimens had mild-to-moderate interstitial fibrosis and tubular atrophy. Regarding the glomerular compartment, median glomerulosclerosis was 10% (IQR 3–28), 7 cases (27%) had focal segmental glomerulosclerosis lesions, and mild mesangial expansion was found in 38% of the biopsy samples (Table 3). Albeit 4 cases presented with endocapillary or extracapillary proliferation, lesions were mild and isolated; hence, their presence was not considered to be proportionate with the degree of AKI severity.

**Table 2:** Pathologic findings in kidney biopsy specimens

Variables	*N =* 26
Underlying nephropathy, *n* (%)	
IgA nephropathy	19 (73)
Probable IgA nephropathy	1 (3.8)
Diabetic nephropathy	2 (7.7)
Diabetic nephropathy + nephrosclerosis	1 (3.8)
Nephrosclerosis	1 (3.8)
Idiopathic nodular glomerulosclerosis	1 (3.8)
None	1 (3.8)

**Table 3:** Histologic features according to underlying nephropathy

Variables	*N =* 26	*n =* 20	*n =* 6
Glomerulus	All patients	IgAN	Non-IgAN diseases
Number of glomeruli per biopsy, Md (IQR)	13 (9–20.5)	12 (9–21.5)	18 (11.5–20.5)
Glomerulosclerosis, Md (IQR), %	10 (3–28)	9 (1–25)	21.5 (3.5–38.7)
Mesangial expansion, *n* (%)			
None (<10%)	6 (23.1)	5 (25)	1 (16.7)
Mild (10%–25%)	10 (38.5)	8 (40)	2 (33.3)
Moderate (26%–50%)	5 (19.2)	4 (20)	1 (16.7)
Diffuse (>50%)	5 (19.2)	3 (15)	2 (33.3)
Endocapillary proliferation*, n* (%)	2 (7.7)	2 (10)	0 (0)
Extracapillary proliferation, *n* (%)	2 (7.7)	2 (10)	0 (0)
Focal segmental glomerulosclerosis lesions, *n* (%)	7 (26.9)	6 (30)	1 (16.7)
Tubulointerstitium			
Interstitial fibrosis and tubular atrophy*, n* (%)			
None (<10%)	0 (0)	0 (0)	0 (0)
Mild (10%–25%)	14 (53.8)	11 (55)	3 (50)
Moderate (26%–50%)	10 (38.5)	9 (45)	1 (16.7)
Severe (>50%)	2 (7.7)	0 (0)	2 (33.3)
RBC casts in tubules, *n* (%)	21 (80.8)	18 (90)	3 (50)
Acute tubular necrosis, *n* (%)	23 (88.5)	17 (85)	6 (100)
Arterio- and arteriolosclerosis	11 (42.3)	8 (40)	3 (50)

IgAN, IgA nephropathy; IQR, interquartile range; Md, median; RBC, red blood cell.

Continuous data are expressed as Md (IQR), categorical variables as n (%).

Direct immunofluorescence study was available in 25 of the 26 cases. Positive (mesangial) IgA staining was observed in 19 samples (73%), most of them 1+ (19%) or 2+ (38%), and C3 staining was positive in 16 specimens (61.5%), predominantly 1+ (46%). Igκ and Igλ were performed in 26.9% (*n =* 7) and 19% (*n =* 5) of the cases, respectively, without detecting light-chain restriction in any case. Only 16 samples were included for electron microscopy, with the most relevant finding being the presence of mesangial deposits in 35% of the specimens.

### Kidney Outcomes

**Figure 1:**
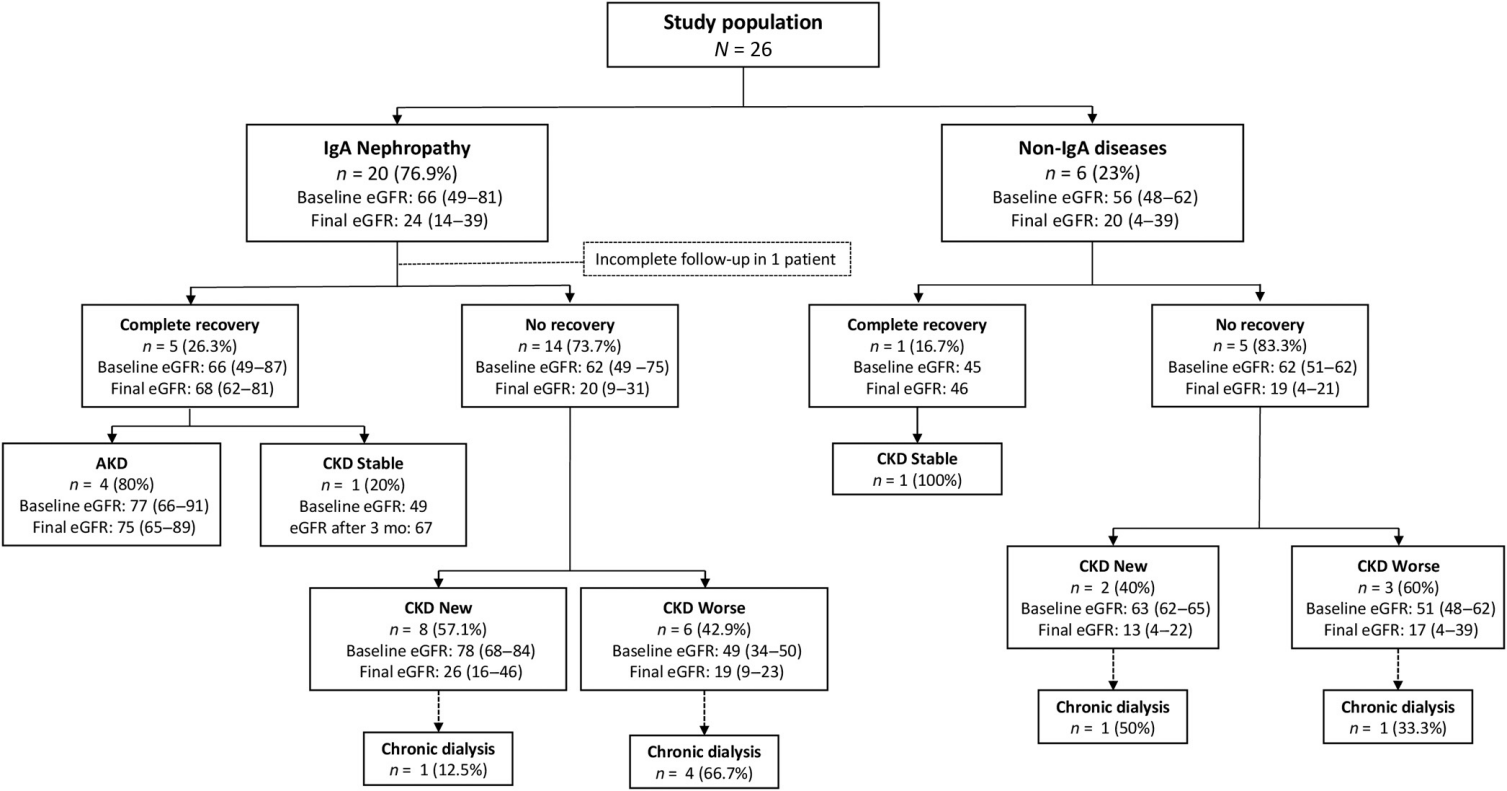
Comprehensive kidney outcomes during follow-up. AKD, acute kidney disease; CKD, chronic kidney disease; eGFR, estimated glomerular filtration rate.

At 12 weeks after hospital discharge, 6 patients (24%) achieved complete kidney function recovery whereas 19 (76%) did not have recovery, of whom 7 (28%) remained dialysis dependent (missing data in 1 case). New CKD developed in 10 cases, whereas CKD worsened in 9 (of 11) cases with previous CKD (Figure 1). Overall, final SCr was 2.2 mg/dl (IQR 11.8–4.5) and final eGFR was 23 ml/min per 1.73 m^2^ (IQR 14–39). At 12 months, 12 (46%) patients had available follow-up data, with a mean SCr of 1.7 mg/dl (IQR 1.3–2.1), mean eGFR of 38 ml/min per 1.73 m^2^ (IQR 31–49), and 7 remained on dialysis.

On the basis of kidney pathology results, subjects with underlying IgAN had a final eGFR of 25 ml/min per 1.73 m^2^ (IQR 14–39) and those with non-IgA diseases of 20 ml/min per 1.73 m^2^ (IQR 4–39). Despite that most patients with previous CKD did not recover, no significant differences were found between the groups based on histologic signs of chronicity, such as glomerulosclerosis, interstitial fibrosis/tubular atrophy, or arterio-arteriolosclerosis (Table 4). Of note, most patients with IgAN received immunosuppression (17 of 20) (Tables 1 and 4), but in 13 of the 17 treated (76.5%), no recovery of kidney function was observed. Although anticoagulation therapy was restarted at discharge in most cases (75%), no AKI relapses were observed during a median follow-up of 10.1 months. None of the patients in whom anticoagulation therapy was restarted underwent a switch from VKAs to DOACs or *vice versa*. A comprehensive description of kidney outcomes is illustrated in Figure 1.

**Table 4:** Differences between patients with complete kidney function recovery and those with no recovery

Variables	*N =* 25^a^	*n =* 6	*n =* 19	*P*
Clinical	All patient**s**	Complete recovery	No recovery	
Sex, *n* (%), male	20 (80)	5 (83.3)	15 (78.9)	1
Age, Md (IQR), yr	76 (63–80)	69 (62–76)	76 (65–82)	0.49
HTN, *n* (%)	23 (92)	5 (83.3)	18 (94.7)	0.43
DM, *n* (%)	10 (40)	1 (16.7)	9 (47.4)	0.34
Chronic kidney disease, *n* (%)	10 (40)	1 (16.7)	9 (47.4)	0.1
SCr, Md (IQR), mg/dl^b^	1.1 (0.9–1.4)	1.1 (0.9–1.1)	1.2 (0.9–1.4)	0.44
eGFR, Md (IQR), ml/min per 1.73 m^2^	62 (49–68)	66 (49–87)	62 (49–75)	0.40
Type of anticoagulant, *n* (%)				0.43
Vitamin K antagonists	23 (92)	5 (83.3)	18 (94.7)	
Direct-acting oral anticoagulants	2 (8)	1 (16.7)	1 (5.3)	
Anticoagulation length, Md (IQR), mo	52 (11–90)	81 (24–93)	48 (11–85)	0.39
INR, Md (IQR)	2.4 (1.6–3.4)	2.0 (1.6–2.6)	2.6 (1.7–3.9)	0.19
AKI stage, *n* (%)				0.23
Stage 1	1 (4)	0	1 (5.3)	
Stage 2	3 (12)	2 (33.3)	1 (5.3)	
Stage 3	21 (84)	4 (66.6)	17 (89.5)	
SCr, Md (IQR), mg/dl	4.1 (2.8–8.3)	3.4 (2.3–8.3)	4.1 (3–8.3)	0.52
Peak SCr, Md (IQR), mg/dl	5.2 (3.9–8.7)	6.1 (2.3–10.6)	7.4 (4.2-9.2)	0.61
Acute dialysis, *n* (%)	11 (44)	2 (33.3)	9 (47.4)	0.66
Gross hematuria, *n* (%)	21 (84)	5 (83.3)	16 (84.2)	1
Anticoagulation withdrawal, *n* (%)	16 (64)	2 (33.3)	14 (73.7)	0.14
Anticoagulation restart (at discharge), *n* (%)	12 (75)	2 (100)	10 (71.4)	1
Immunosuppressive treatment (for AKI), *n* (%)	18 (72)	5 (83.3)	13 (68.4)	0.64
Corticosteroids, *n* (%)	18 (72)	5 (83.3)	13 (68.4)	1
Mycophenolic acid, *n* (%)	5 (20)	0	5 (26.3)	0.27
Histopathologic^6^^,^^7^^,^^c^				
IgAN vs. non-IgAN	19/6	5/1	14/5	1
Glomerulosclerosis (0–3)	1 (0–2)	1 (0.5–1)	0 (0–2)	0.28
IFTA (0–6)	3 (2–4)	3 (2–4)	3 (2–4)	1
Arterio- or arteriolosclerosis (absent vs. present)	10/11	3/1	7/10	0.31
Total chronicity score (0–10)	4 (3–5)	4.5 (3–5)	4 (3–5)	0.72

AKI, acute kidney injury; DM, diabetes mellitus; eGFR, estimated glomerular filtration rate; HTN, hypertension; IFTA, Interstitial Fibrosis and Tubular Atrophy; IgAN, IgA nephropathy; INR, international normalized ratio; IQR, interquartile range; Md, median; MDRD, Modification of Diet in Renal Disease; SCr, serum creatinine.

Continuous data are expressed as Md (IQR), categorical variables as n (%).eGFR, calculated by MDRD study equation.

a 1 patient excluded (incomplete follow-up).

b SCr available in the last 12 weeks before admission.

c 22 of 26 biopsies were centrally evaluated.

### Review of the Literature

We found 3 case series (*n =* 63)^1^^,^^3^^,^^4^ and 29 case reports describing histologic findings in patients with ARN.^2^^,^^9^, ^10^, ^11^, ^12^, ^13^, ^14^, ^15^, ^16^, ^17^, ^18^, ^19^, ^20^, ^21^, ^22^, ^23^, ^24^, ^25^, ^26^, ^27^, ^28^, ^29^, ^30^, ^31^, ^32^, ^33^, ^34^, ^35^ Of 92 cases, 37 (40%) were compatible with IgAN. The main characteristics and outcomes of patients with ARN and IgAN available in the literature are summarized in Supplementary Table S1.

## Discussion

In this study of patients with AKI and biopsy-proven ARN, we found that the most common underlying kidney disease was IgAN. Furthermore, we confirmed previous observations indicating the occurrence of ARN is exceptional in the absence of previous or concomitant nephropathy.^1^^,^^4^ Similar to preceding series, kidney survival was poor, with almost one-third of the patients remaining on chronic dialysis at the end of follow-up.

To the best of our knowledge, at the present time, only 3 case series (*n =* 63)^1^^,^^3^^,^^4^ and 29 case reports have described histologic information on patients with ARN.^2^^,^^9^, ^10^, ^11^, ^12^, ^13^, ^14^, ^15^, ^16^, ^17^, ^18^, ^19^, ^20^, ^21^, ^22^, ^23^, ^24^, ^25^, ^26^, ^27^, ^28^, ^29^, ^30^, ^31^, ^32^, ^33^, ^34^, ^35^ Of note, 37 of 92 (40%) cases were compatible with IgAN, comprising by far the most common underlying kidney disease associated to ARN. Moreover, in the largest series published to date of biopsy-proven ARN, IgA-predominant immune complexes in the glomeruli were observed in 34% of the patients (without specifying how many of them met IgAN criteria), representing the most frequent histologic lesion in kidney biopsies. This is in line with our findings, in which most of the cases had confirmed IgAN or presented histologic features suggestive of IgAN (mesangial expansion in light microscopy plus mesangial deposits in electron microscopy). Notably, it is plausible that the high percentage of cases with IgAN in our series is related to the moderately high incidence and prevalence of IgAN in Spain. In a retrospective study of >20,000 kidney biopsies recorded in the Spanish Registry of Glomerulonephritis, the prevalence of IgAN ranged between 12.8% and 15.5%, with IgAN representing 23.8% to 26.7% of the total number of kidney biopsies with a diagnosis of glomerulonephritis.^36^

Some studies have suggested that the incidence of IgAN is increasing in older adults,^36^ although the explanations for this finding are unknown. In a retrospective multicentric study of elderly patients with IgAN, our group described that the most frequent clinical presentation was AKI associated with hematuria. Interestingly, 34% of the patients were taking anticoagulant drugs and the number increased up to 42% among those aged >72 years. Moreover, the number of patients receiving anticoagulant agents was significantly higher in those with AKI associated with hematuria compared with the other groups.^37^ The latter findings suggest that the use of anticoagulants in subjects with preexisting or underlying IgAN might be a trigger for the development of macrohematuria, thus, leading to diagnosis of the disease in elderly patients.

Data on the clinical importance of ARN are scarce. In a recent study, among 8636 native kidney biopsy samples reviewed at the Ohio State University Wexner Medical Center, only 41 cases (0.5%) had deterioration in kidney function that could be related to anticoagulation.^3^ Another retrospective study in 126 allograft biopsies from transplant recipients on long-term anticoagulation (minimum of 2 years follow-up) reported that only 1 case presented features of ARN.^2^ Hence, it seems that ARN is an infrequent diagnosis in daily clinical practice. Conversely, other studies have reported that patients with CKD who are receiving warfarin have a 95% CI for incident AKI of 6.8% to 26% per year.^38^ Nonetheless, the absence of histologic data in most retrospective cohort studies precludes determining whether all AKI cases were actually secondary to ARN. Furthermore, according to a systematic review and meta-analysis based on 4 cohort studies, prevalence of ARN ranged from 19% to 63%; notwithstanding, these results were in the context of high heterogeneity.^39^ It is important to consider that reluctance to perform kidney biopsies in patients on anticoagulation therapy prevents from drawing conclusions on the real epidemiology of ARN.

Traditionally, the diagnosis of ARN can be based on a SCr level increase of ≥0.3 mg/dl in patients with an INR >3 in the previous week, with no other evident cause, even in the absence of a kidney biopsy.^40^ Nevertheless, moderate overanticoagulation may be sufficient to develop ARN.^2^ This could explain why our study population presented AKI despite a median INR of 2.4 at admission. It should be noted that an important limitation of our work is the absence of available INR levels before the AKI episode. Nevertheless, the proportion of warfarin-treated patients is currently decreasing in favor of DOACs. In fact, dabigatran is currently the most often reported cause of ARN after warfarin.^41^ Therefore, the inclusion of INR levels for the diagnosis of ARN is likely to lose value in time.

The pathophysiology of AKI-associated ARN is not completely understood. The absence of retrodiffusion of Tamm-Horsfall protein into the glomeruli does not support a pure obstructive hypothesis secondary to intratubular RBC or hemoglobin casts.^42^^,^^43^ Yet, some authors propose that ARN should be considered when the number of RBC casts is disproportionate to the severity of glomerular changes in patients under anticoagulation drugs or with acute coagulopathy.^3^ Other pathogenic mechanisms may be related to the direct tubular toxicity of hemoglobin or other molecules released from the RBC. Hemoglobin and its heme-derivates induce oxidation, mitochondrial damage, inflammation, fibrosis, and cell death.^44^^,^^45^ Preclinical studies in rats suggest that thrombin plays an important role in the glomerular filtration barrier function because inhibition of protease-activated receptor 1 resulted in a clinicopathologic picture similar to that of ARN.^46^ Notably, anticoagulation with warfarin induced AKI in 5/6 nephrectomy rats in a dose-dependent manner as compared with control rats, and histologic evidence of acute tubular injury with RBCs and RBC casts was observed.^47^ The multiple mediators thought to contribute to hematuria-induced kidney injury have been reviewed elsewhere.^45^

Management of ARN at the present time remains largely supportive. In the aforementioned study in 5/6 nephrectomy rats, treatment with vitamin K prevented AKI and histologic changes associated with warfarin.^47^ Nevertheless, stopping anticoagulation in some cases may be problematic (high-risk atrial fibrillation, mechanical heart valves, pulmonary embolism) and reversing acute coagulopathy (disseminated intravascular coagulopathy) may be challenging. Furthermore, there is no evidence that reversal of anticoagulation limits kidney injury in humans. Of note, although anticoagulation withdrawal was performed in 61% of our study population, kidney function outcome was similar than in those in whom anticoagulant treatment was maintained. Recent studies have proposed that the use of DOACs may be associated with lower risks of adverse renal outcomes than warfarin.^48^^,^^49^ In a Canadian population-based cohort study in >20,000 elderly adults with atrial fibrillation, the use of DOACs (dabigatran, rivaroxaban, apixaban) was associated with a significantly lower risk of AKI compared with warfarin, and the risk was consistent across each eGFR strata.^50^ The latter suggest that the use of DOACs in patients with multiple risk factors for developing ARN may be an attractive preventive strategy. In addition, it is important to note that approximately 20% of our patients had significant microhematuria several months before the AKI event, advocating that urinary sediment should be closely monitored in those patients with risk factors for ARN in whom anticoagulant therapy is initiated.

Considering most cases of ARN have an underlying glomerular disease (mostly IgAN), the use of immunosuppressive drugs may seem appealing. Since most patients in our series were diagnosed with ARN retrospectively, the use of immunosuppressants can be explained by the fact that local practice in some centers includes immunosuppressive therapy for hematuria-related AKI in the context of IgAN;^37^ notwithstanding only supportive treatment is currently recommended.^51^ In the present study, the use of immunosuppressive treatment (prednisone, mycophenolic acid) for ARN had no influence on renal prognosis after 3 months of follow-up. On the other hand, bearing in mind the renal accumulation of iron in this pathologic setting, therapies for iron chelation or the use of antioxidants to ameliorate heme-mediated oxidative stress appears as promising therapeutic targets for ARN.^52^, ^53^, ^54^

Kidney outcomes of patients with ARN are poor. In the original study of Brodsky *et al.*,^1^ 6 of 9 patients did not recover from AKI and 4 remained on dialysis. Also, in a French multicenter case study, 9 of 13 patients progressed to CKD, 1 remained on dialysis, and only 1 case fully recovered.^4^ In addition, diagnosis of ARN has been associated with an increased mortality rate according to several reports.^39^^,^^55^ These findings are similar to ours, where only 24% of patients achieved complete recovery and 28% remained dialysis dependent, confirming the adverse renal prognosis of patients with biopsy-proven ARN. Unexpectedly, no correlation was found between histologic signs of chronicity and kidney outcomes, probably owing to the relatively small population included in our study. In a few cases, non-significant proliferative and focal segmental glomerulosclerosis lesions were encountered. The latter has also been observed in previous series.^3^

Data on factors associated to kidney recovery in patients with ARN are scarce. In our case, we did not find significant differences between patients with complete kidney function recovery and those with no recovery. Some studies have described that the presence of atrial fibrillation and higher albumin levels were protective against the development of ARN.^39^^,^^56^

The main limitation of our study is its observational and retrospective nature. Nevertheless, the present work represents one of the largest series with detailed histologic description of patients with ARN and a relatively long follow-up period.

In summary, ARN is an uncommon clinical entity that should be suspected in patients on anticoagulant therapy or with acute coagulopathy who present with AKI and hematuria. Our findings confirm that subjects with mild or very mild forms of IgAN are at increased risk for the development of ARN and that IgAN represents the most common underlying kidney disease associated to ARN. Nonetheless, it is important to consider that IgAN is the most common primary glomerulonephritis and is typically manifested by hematuria. Hence, ARN may be easier to detect in these cases. Considering the poor renal prognosis, new therapeutic approaches are urgently needed, including preventive strategies in patients with risk factors for developing ARN. Larger observational or prospective studies that include histologic descriptions should be performed to get a better understanding of the true epidemiology of ARN.

## Disclosure

All the authors declared no competing interests.

## Supplementary Material

Supplementary File (PDF)

**Table S1.** Clinical and histologic characteristics and outcomes of patients with ARN and IgAN available in the literature.
