# Supplementary material for: IgA Nephropathy Is the Most Common Underlying Disease in Patients With Anticoagulant-Related Nephropathy
Source: Kidney Int Rep. 2022 Jan 19;7(4):831–40. doi: 10.1016/j.ekir.2022.01.1048 (PMC9039479; doi:10.1016/j.ekir.2022.01.1048)
Supplement: Supplementary File (PDF) [file mmc1.pdf]

IgA nephropathy is the most common underlying disease in patients with anticoagulant-related nephropathy

Index to Supplemental Material

1. Supplemental Table S1..2

Supplemental Table S1. Clinical and histologic characteristics and outcomes of patients with ARN and IgAN available in the literature.

Author (year)	Number of patients	Age (years)	Sex	Anticoagulant	Presentation	INR	Baseline SCr (mg/dl)	Peak SCr (mg/dl)	Underlying kidney disease	Histopathologic findings	Management	Outcome
August (2002)	1	59	F	Phenprocoumon	Gross hematuria, edema, dyspnea	3.6	1	8.4	IgAN	Mesangial expansion, RBC	HD, steroids	Recovery
Brodsky (2009)	9	61+/-19	M: 5<br>F: 4	Warfarin	Hematuria (n=9)	4.3+/-1.9	1.3+/-0.8	4.2+/-2.3	IgAN (n=2), IgAN/DN (n=1), SLE (n=1), ICGN (IgG) (n=1), FSGS (n=1), NS (n=2)	Mesangial expansion (n=2) RBC (n=4), ATN (n=9), IgA mesangial deposits in IF (n=5)	N/A	Recovery (n= 4), HD (n=3), death (n=1), CKD (n=1)
Cleary (2010)	1	51	F	Acenocoumarol	Gross hematuria, proteinuria, AKI	6.2	0.8	8.6	IgAN	Mesangial expansion, RBC, crescent and necrotizing lesions	Steroids, cyclophosphamide	Recovery
Woo (2012)	1	48	M	Warfarin	Hematuria, proteinuria	>3	N/A	2	IgAN	Mild mesangial IgA deposits, RBC	N/A	N/A
Moeckel (2013)	1	67	M	Dabigatran	Gross hematuria	1.6	1	5.5	IgAN	Moderate mesangial IgA deposits, RBC	Fluid therapy	CKD
Escoli (2015)	1	69	F	Dabigatran	Hematuria, AKI	2.3	1.5	8	IgAN	Moderate mesangial IgA deposits, RBC, ATN	Fluid therapy, HD	CKD
Ng (2016)	1	56	F	Warfarin	Hematuria, AKI	5	0.8	3.5	IgAN	FSGS, RBC, ATN	Steroids	CKD
Góis (2017)	1	84	M	Acenocoumarol	Gross hematuria, AKI	2	1	4.6	IgAN	Mild mesangial hypercellularity, moderate IgA deposits, RBC, ATN	Anticoagulant stopped	CKD
Kalaitzidis (2017)	1	78	F	Dabigatran	Hematuria, AKI	1.9	1.1	6.8	IgAN	Moderate mesangial IgA deposits, RBC, ATN	Anticoagulant stopped	Recovery
Golbin (2017)	13	69+/-13	M: 10<br>F: 3	Fluindione (n=7), acenocoumarol (n=2), warfarin (n=4)	Gross hematuria (n=13)	5.7+/-3	5.5<br>HD (n=6)	eGFR 77+/-25 ml/min	IgAN (n=5), IgAN + DN (n=1), HSP (n=1), PIGN (n=1), ICGN (IgG k) (n=1), HTNS (n=3), NI (n=1)	RBC (n=11), ATN (n=12), fibrosis (n=9), IgA mesangial deposits in IF (n=6)	Anticoagulant stopped	Recovery (n=1), CKD (n=9), HD dependent at 1 yr (n=1), death (n=2)
Brodsky (2017)	1	82	F	Apixaban	Oliguria, microscopic hematuria, AKI	N/A	3.2	8.5	Focal and segmental necrotizing and crescentic GN (active)	Small cellular crescent, AKI, RBC, IgA mesangial deposits in IF	Steroids	HD dependent

Ishii (2018)	1	55	M	Warfarin	Hematuria, proteinuria, AKI	3.7	0.7	9	IgAN	Mild mesangial hypercellularity, moderate IgA deposits, RBC, crescentic lesions	Fluid therapy	CKD
Brodsky (2019)	41	62+/-14	M: 26<br>F: 15	Warfarin (n=28), heparin (n=4), apixaban (n=2), clopidogrel and aspirin (n=1), coagulopathy (n=6)	Hematuria (n=41)	5.6+/-6	1.2+/-0.4	4.3+/-1.9	IgAN (n=14), ICGN (IgG) (n=5), SLE (n=3), PIGN (n=8), FSGS (n=4), DN (n=2), other (n=5)	Mild immune complex deposition (n=22), RBC (n=22), focal-mild interstitial inflammation (n=22)	N/A	N/A
Li X (2019)	1	61	M	Dabigatran	Gross hematuria, AKI	4	0.9	10.1	IgAN	Mesangial expansion, RBC, ATN, crescent and necrotizing lesions, moderate IgA deposits	HD, steroids	Recovery
Li J (2019)	1	70	M	Warfarin	Hematuria, proteinuria, AKI	8.7	1.3	4.4	IgAN	Mesangial expansion, RBC, moderate IgA deposits	Fluid therapy, HD	CKD
Rawala (2019)	1	61	M	Warfarin	Gross hematuria, AKI	3.5	2.0	6.8	IgAN	Mild mesangial hypercellularity, mild IgA deposits, RBC	Steroids, HD	HD dependent
Ikeda (2019)	1	67	F	Dabigatran	Gross hematuria, proteinuria, AKI	2.4	0.5	3.6	IgAN	Moderate IgA deposits, RBC, ATN	Anticoagulant stopped	Recovery

Abbreviations: AKI: acute kidney injury; ARN: anticoagulant-related nephropathy; ATN: acute tubular necrosis; CKD: chronic kidney disease; DN: diabetic nephropathy; eGFR: estimated glomerular filtration rate; F: female; FSGS: focal segmental glomerulosclerosis; HD: hemodialysis; HSP: Henoch-Schönlein purpura; HTNS: hypertensive nodular sclerosis; ICGN: immune-complex glomerulonephritis; IF: immunoflourescense; IgAN: IgA nephorpathy; N/A: not available; INR: International Normalized Ratio; M: male; NI: not identified; NS: nodular sclerosis; PIGN: pauci-immune glomerulonephritis; RBC: red blood cell casts; SCr: serum creatinine; SLE: systemic lupus erythematous;
